# Prediction of network pharmacology and molecular docking-based strategy to determine potential pharmacological mechanism of Liuwei Dihuang pill against tinnitus

**DOI:** 10.1097/MD.0000000000031711

**Published:** 2022-11-18

**Authors:** Zhongbiao Wu, Zhongyan Zhu, Jian Cao, Weikun Wu, Shiping Hu, Chengcheng Deng, Qiang Xie, Xinmei Huang, Chengkun You

**Affiliations:** a Jiangxi Hospital of Integrated Traditional Chinese and Western Medicine, Nanchang, Jiangxi, China; b Affiliated Hospital of Jiangxi University of Traditional Chinese Medicine, Nanchang, Jiangxi, China; c Jiangxi University of Traditional Chinese Medicine, Nanchang, Jiangxi, China; d Pinghu Hospital of Traditional Chinese Medicine, Jiaxing, Zhejiang, China.

**Keywords:** Liuwei Dihuang Pill, molecular docking, network pharmacology, tinnitus

## Abstract

Liuwei Dihuang Pill is widely used to treat tinnitus in China. However, the underlying mechanism of Liuwei Dihuang Pill in treating tinnitus still remains unclear.

**Objective:**

To explore the potential pharmacological mechanism of Liuwei Dihuang Pill in the treatment of tinnitus based on network pharmacology and molecular docking.

**Methods:**

The active components of the Liuwei Dihuang Pill were obtained from the traditional Chinese medicine systems pharmacology database and analysis platform (TCMSP) database. Cytoscape software was used to draw the active component-target network diagram of Liuwei Dihuang Pill, and obtain the core components. Then the corresponding targets were also obtained from the TCMSP database. Targets related to tinnitus were obtained from the GeneCards, DisGeNET, TTD and DrugBank databases. The String database was used to construct protein–protein interaction (PPI) network of common targets of drugs and diseases, then the core targets were screened out. The Annotation, Visualization and Integrated Discovery (DAVID) database was used for gene ontology (GO) enrichment and Kyoto encyclopedia of genes and genomes (KEGG) pathway analysis of common targets. Finally, the molecular docking between the core component and the core target was carried out by AutoDock.

**Results:**

The core components of Liuwei Dihuang Pill in the treatment of tinnitus including quercetin, stigmasterol, kaempferol, β-sitosterol, tetrahydroalstonine, which may act on core targets such as STAT3, transcription factor AP-1 (JUN), tumor necrosis factor (TNF), interleukin-6 and MAPK3. HIF-1 signaling pathway, Influenza A, P53 signaling pathway, and Toll-like receptor signaling pathway play a role in anti-inflammatory, improving microcirculation in the blood-labyrinth barrier, increasing cochlear blood flow, and preventing hair cell damage. The molecular docking results showed that the affinity between core components and core targets was good.

**Conclusion:**

The potential mechanism of Liuwei Dihuang Pill in the treatment of tinnitus was preliminarily discussed in this study, which may provide a theoretical basis and evidence for further experimental research.

## 1. Introduction

Tinnitus is the absence of a corresponding external sound source or electrical stimulation, but subjectively there is a sound in the ear or brain.^[1]^ The prevalence of tinnitus ranged from 4.3% to 51.33%, but varied with age and sex. The highest increase in prevalence from the previous decade in age occurs during the fifth and sixth decades, and the highest prevalence occurred in the seventh decade at 34.27%.^[2]^ Until now, the pathological mechanisms of tinnitus have not been clearly elucidated,^[3]^ but the main pathogenesis of tinnitus is now associated with abnormal neuronal firing, mechanical dysfunction of the cochlea, abnormal micromechanical activity of the cochlea, mechanical feedback of the cochlea, and abnormal vibration of outer hair cells.^[4,5]^Obviously, the deeper mechanism of tinnitus needs to be studied from various aspects and from a broad perspective, rather than being limited to lesions in the auditory conduction pathway. While there is currently no golden standard treatment for tinnitus, counseling, psychotherapy, pharmacological approaches, masking devices, individualized sound stimulation, and cognitive behavioral therapy are the most widely used strategies.^[6]^ Its mechanism of action may be related to the following factors, including enhances cochlear blood flow，protects against ototoxic stimuli and restrains apoptosis，impedes age-associated degenerative processes in the inner ear，affects neurotransmitter actions，and so on.^[7]^ While some of these treatment modalities are effective in reducing awareness of tinnitus or related distress, they can be time-consuming, expensive, and not covered by insurance, so are only available to a limited population.^[8]^

Liuwei Dihuang Pill originated in Key to Therapeutics of Children’s Disease which was written by Qian Yi in Song Dynasty. Liuwei Dihuang pill includes 6 traditional Chinese medicines, including Radix Rehmanniae Preparata, Rhizoma Dioscoreae, Fructus Corni, Poria, Rhizoma Alismatis and Cortex Moutan, which has the function of nourishing kidney Yin. There includes complementary reinforcing and reducing methods in the whole prescription, which play a role in nourishing kidney yin and improving ear function.^[9]^ Liuwei Dihuang Pill is often used to treat tinnitus. Besides, Liuwei Dihuang pill can also be used to treat a variety of other diseases, including type 2 diabetes,^[10]^ postmenopausal osteoporosis,^[11]^ triple negative breast cancer^,[12]^ and Primary nephrotic syndrome.^[13]^ Modern scientific research had confirmed the function of Liuwei Dihuang pill manifested in anti-inflammation,^[14]^ Inhibition of apoptosis,^[15]^ and so on. Numerous papers have shown that Liuwei Dihuang pill has a good clinical effect on tinnitus.^[16,17]^ However, the effects and mechanism of Liuwei Dihuang Pill of tinnitus were not clear.

**Figure 1.**
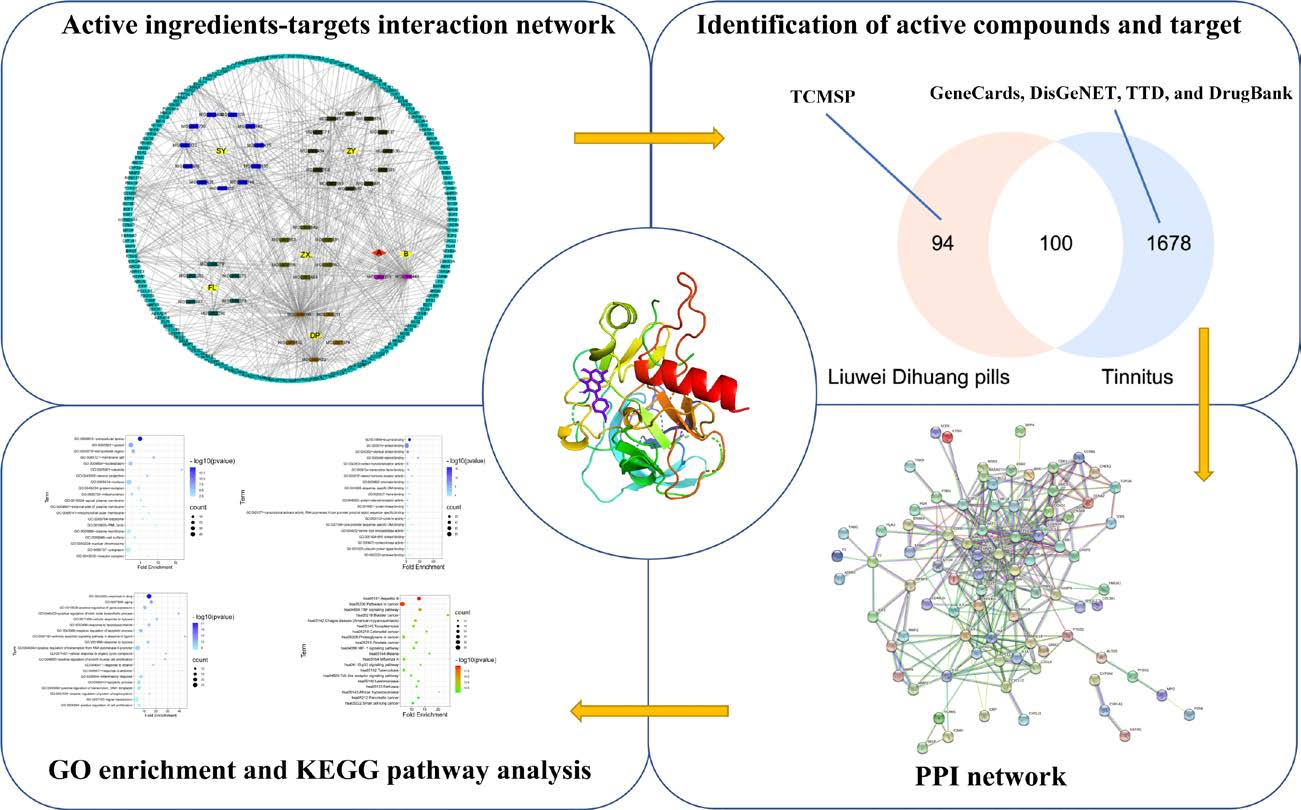
Graphical abstract.

Network pharmacology is a emerging interdisciplinary fields, which based on the theory of systems biology, and integrates computer science with bioinformatics, Network pharmacology can be used to analyze the “multi-component, multi-target, multi-pathway” synergistic relationship between drugs, diseases and targets. It has played an important role in explaining the pharmacological mechanism of traditional Chinese medicine, exploring the toxicity mechanism of traditional Chinese medicine, and researching and developing new traditional Chinese medicine.^[18]^ Molecular docking is based on the receptors and ligands with known structures, in accordance with the 3 complementary principles of geometry, energy, and chemical environment, to identify the interaction between molecules, and to predict the best binding mode between molecules. It has important value and potential advantages in the exploration of the potential target and action mechanism of active ingredients in traditional Chinese medicine, and the study of the pharmacological mechanism of prescription of Chinese herbal compound.^[19]^ This study aims to explore the potential mechanism of Liuwei Dihuang Pill in the treatment of tinnitus using traditional Chinese medicine network pharmacology and molecular docking methods, to provide a theoretical basis for further research. The graphical abstract was shown as follow (Fig. 1).

## 2. Materials and methods

### 2.1. Materials

Databases: Traditional Chinese Medicine Systems Pharmacology Database and Analysis Platform (TCMSP, https://tcmspw.com/index.php),^[20]^ Uniprot database (http://www.uniprot.org),^[21]^ GeneCards database (https://www.genecards.org), DisGeNET database (https://www.disgenet.org), TTD database (http://bidd.nus.edu.sg/group/cjttd), DrugBank database (https://www.drugbank.ca),^[22]^ String database (Version 11.0, https://string-db.org),^[23]^ the Annotation, Visualization and Integrated Discovery (DAVID) database (https://david.ncifcrf.gov),^[24]^ PDB database (https://://www.rcsb.org). Online drawing tools: Venny (Version 2.1.0, https://bioinfogp.cnb.csic.es/tools/venny), Bioinformatics (http://www.bioinformatics.com.cn). Software: Cytoscape software (version 3.8), AutoDock software (version 4.2.6), AutoDock Tools software (version 1.5.6), PyMOL software (version 2.4.0).

### 2.2. Screening of compositions and targets in Liuwei Dihuang pill

The chemical compositions of Radix Rehmanniae Preparata, Rhizoma Dioscoreae, Fructus Corni, Poria, Rhizoma Alismatis and Cortex Moutan were collected from the TCMSP database, and the active ingredients were screened according to ADME (Adsorption, Distribution, Metabolism, Excretion), meeting the 2 conditions of oral bioavailability (OB) ≥ 30 % and drug-like (DL) ≥ 0.18. After the active ingredients of each drug were obtained, the corresponding targets were also searched in the TCMSP database, and the target name was standardized by the Uniprot database. Cytoscape software was used to draw the active components-targets network diagram of Liuwei Dihuang Pill, and the topological analysis was carried out through the network analyzer function of the software. The core components of Liuwei Dihuang Pill were obtained according to the degree value.

### 2.3. Screening of tinnitus-related targets

Tinnitus-related targets were retrieved from the GeneCards, DisGeNET, TTD, and DrugBank databases using “tinnitus” (Mesh) as the key search term. After merging the targets obtained from the above database, as well as removing duplicates, the tinnitus-related targets were obtained.

### 2.4 Construction of a protein–protein interaction (PPI) network between interaction targets and acquisition of core targets

The interaction targets of Liuwei Dihuang pill and tinnitus were obtained through Venny, and then the Venn diagram was drawn. The PPI network between interaction targets was built using the String database. The mode was “Multiple Protenin,” and the organism was “Homo sapiens.” The minimum required interaction score was set to “Highest confidence (0.900),” and the disconnected nodes in the network were hidden. Other parameters remained unchanged. The obtained node1, node2, and combined score were imported into Cytoscape software for visual analysis. The network analyzer function of the software was used for topological analysis, and the core targets of Liuwei Dihuang Pill in the treatment of tinnitus was obtained according to the degree value.

### 2.5. *GO enrichment and* Kyoto encyclopedia of genes and genomes (KEGG) *pathway analysis*

Gene Ontology (GO) enrichment and KEGG pathway analysis were performed on the interaction targets of Liuwei Dihuang Pill and tinnitus using DAVID database. The Select Identifier was set to “OFFICIAL GENE SYMBOL” and the species and background were set to “Homo sapiens.” Then the cell component (CC), molecular function (MF), biological process (BP), and KEGG pathway analysis were performed. The data was exported and sorted according to the *P* value. For each GO enrichment and KEGG pathway, 20 items with the lowest *P* value were selected to draw the advanced bubble diagram by Bioinformatics.

### 2.6. Construction of targets-pathways interaction network

The 20 items with the lowest *P* value in the KEGG pathways were imported into Cytoscape software, and the interaction targets between the compounds and disease targets included in the above KEGG pathways were also imported into the software, then the targets-pathways interaction network was constructed.

### 2.7. Molecular docking

AutoDock molecular docking was performed between the selected core components and the core targets. The mol2 formats of the core components were downloaded from the TCMSP database, and transformed into pdb formats by using PyMOL, then saved as pdbqt formats by using AutoDock Tools. The pdb formats of the core targets was downloaded from the PDB database (the protein complex with ligand and resolution of < 3A was selected), and the water removal, hydrogenation, and removal of the original ligand were performed by PyMOL. The atomic type was set as Assign AD4 type, and then imported into AutoDock Tools to save as pdbqt formats. The spatial position of the original ligand in the protein complex was defined as the active pocket, and the Lamarckian genetic algorithm was selected to run molecular docking by AutoDock. The binding free energy was used to screen the best docking results. Finally, the results were visualized by PyMOL.

## 3. Results

### 3.1. Obtainment of components and targets in Liuwei Dihuang pill

**Figure 2.**
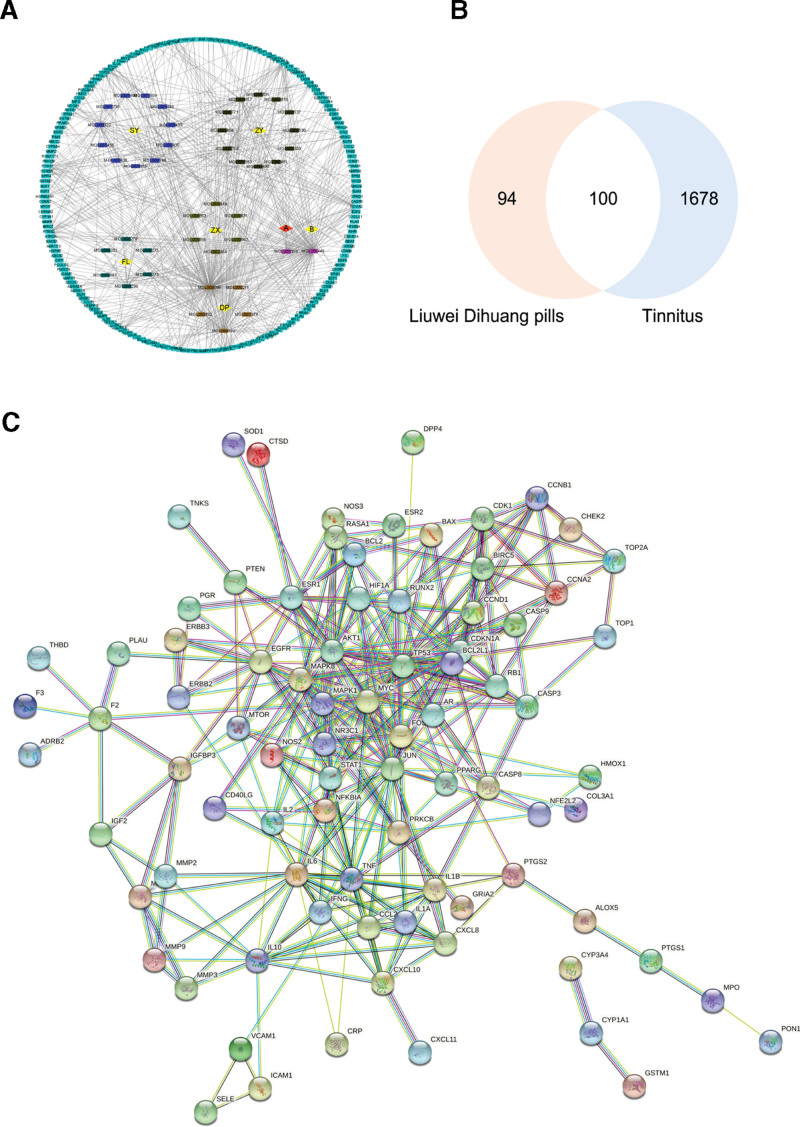
(A) Active ingredients-targets interaction network diagram of Liuwei Dihuang Pill. (B) Venn diagram of Liuwei Dihuang pill-tinnitus-related targets. (C) PPI network. PPI = protein–protein interaction.

The components and targets of Liuwei Dihuang Pill were obtained from the TCMSP database, and OB ≥ 30% and DL ≥ 0.18 were used as the included criteria. After eliminating the non-target components and removing the duplication, 42 active components were obtained. Among them, there are no unique ingredients of Radix Rehmanniae Preparata, 11 unique ingredients of Rhizoma Dioscoreae, 12 unique ingredients of Fructus Corni, 6 unique ingredients of Poria, 6 unique ingredients of Rhizoma Alismatis, and 5 unique ingredients of Cortex Moutan. Besides, there was 1 common ingredient of Radix Rehmanniae Preparata, Rhizoma Dioscoreae, and Fructus Corni. There was 1 common ingredient of Radix Rehmanniae Preparata, Rhizoma Dioscoreae, Rhizoma Alismatis, Fructus Corni, and Cortex Moutan. The 42 active components were arranged according to the OB value from large to small, and the top 20 components were shown (Table 1). By retrieving the TCMSP database, a total of 30 targets of Radix Rehmanniae Preparata, 75 targets of Rhizoma Dioscoreae, 69 targets of Fructus Corni, 23 targets of Poria, 5 targets of Rhizoma Alismatis and 163 targets of Cortex Moutan were searched. Repetitive values were removed, and the Uniprot database was used to standardize the names of targets. Then a total of 194 targets of Liuwei Dihuang pill were obtained. Cytoscape software was used to draw the active components-targets interaction network of Liuwei Dihuang Pill (Fig. 2A). The top 5 core components of Liuwei Dihuang Pill were quercetin, stigmasterol, kaempferol, β-sitosterol, and tetrahydroalstonine according to the degree value of the network analyzer function of the software.

**Table 1:** Top 20 active components of Liuwei Dihuang pill.

MOL ID	Name of active ingredient	OB(%)	DL
MOL000546	diosgenin	80.88	0.81
MOL005531	Telocinobufagin	69.99	0.79
MOL001736	(-)-taxifolin	60.51	0.27
MOL005430	hancinone C	59.05	0.39
MOL000211	Mairin	55.38	0.78
MOL000492	(+)-catechin	54.83	0.24
MOL000322	Kadsurenone	54.72	0.38
MOL000098	quercetin	46.43	0.28
MOL001495	Ethyl linolenate	46.1	0.2
MOL005465	AIDS180907	45.33	0.77
MOL000449	Stigmasterol	43.83	0.76
MOL005440	Isofucosterol	43.78	0.76
MOL002879	Diop	43.59	0.39
MOL000282	ergosta-7,22E-dien-3beta-ol	43.51	0.72
MOL007374	5-[[5-(4-methoxyphenyl)-2-furyl]methylene]barbituric acid	43.44	0.3
MOL001494	Mandenol	42	0.19
MOL000422	kaempferol	41.88	0.24
MOL000283	Ergosterol peroxide	40.36	0.81
MOL005503	Cornudentanone	39.66	0.33
MOL000275	trametenolic acid	38.71	0.8

### 3.2. Acquisition of tinnitus-related targets

A total of 1733 tinnitus-related targets were obtained in the GeneCards database, 103 tinnitus-related targets were obtained in the DisGeNET database, 1 tinnitus-related target was obtained in the TTD database and 3 tinnitus-related targets were obtained in the DrugBank database. After merging the targets obtained from the 4 databases and deleting the duplicate values, 1778 tinnitus targets were obtained.

### 3.3 Construction of a PPI network between interaction targets and acquisition of core targets

There were 100 interaction targets of Liuwei Dihuang pill and tinnitus were obtained through Venny (Fig. 2B). Importing 100 interaction targets into the String database to build PPI networks (Fig. 2C) (the disconnected nodes in the network were hidden). The obtained node1, node2, and combined score were imported into Cytoscape software for visual analysis. The network analyzer function of the software was used for topological analysis. According to the degree value, the core targets of Liuwei Dihuang Pill in the treatment of tinnitus were TP53, transcription factor AP-1 (JUN), MAPK1, AKT1 and tumor necrosis factor (TNF).

### 3.4. GO enrichment and KEGG pathway analysis

The 100 interaction targets of Liuwei Dihuang Pill and tinnitus were imported into the DAVID database for GO enrichment and KEGG pathway analysis. A total of 41 cellular components (CC), 82 molecular functions (MF), 456 biological processes (BP), and 108 KEGG pathways were obtained. The data was exported and sorted according to the *P* value. For each GO enrichment and KEGG pathway, 20 items with the lowest *P* value were selected to draw the advanced bubble diagram by Bioinformatics. The darker the bubble, the smaller the *P* value, and the larger the bubble, the greater the number of genes.

**Figure 3.**
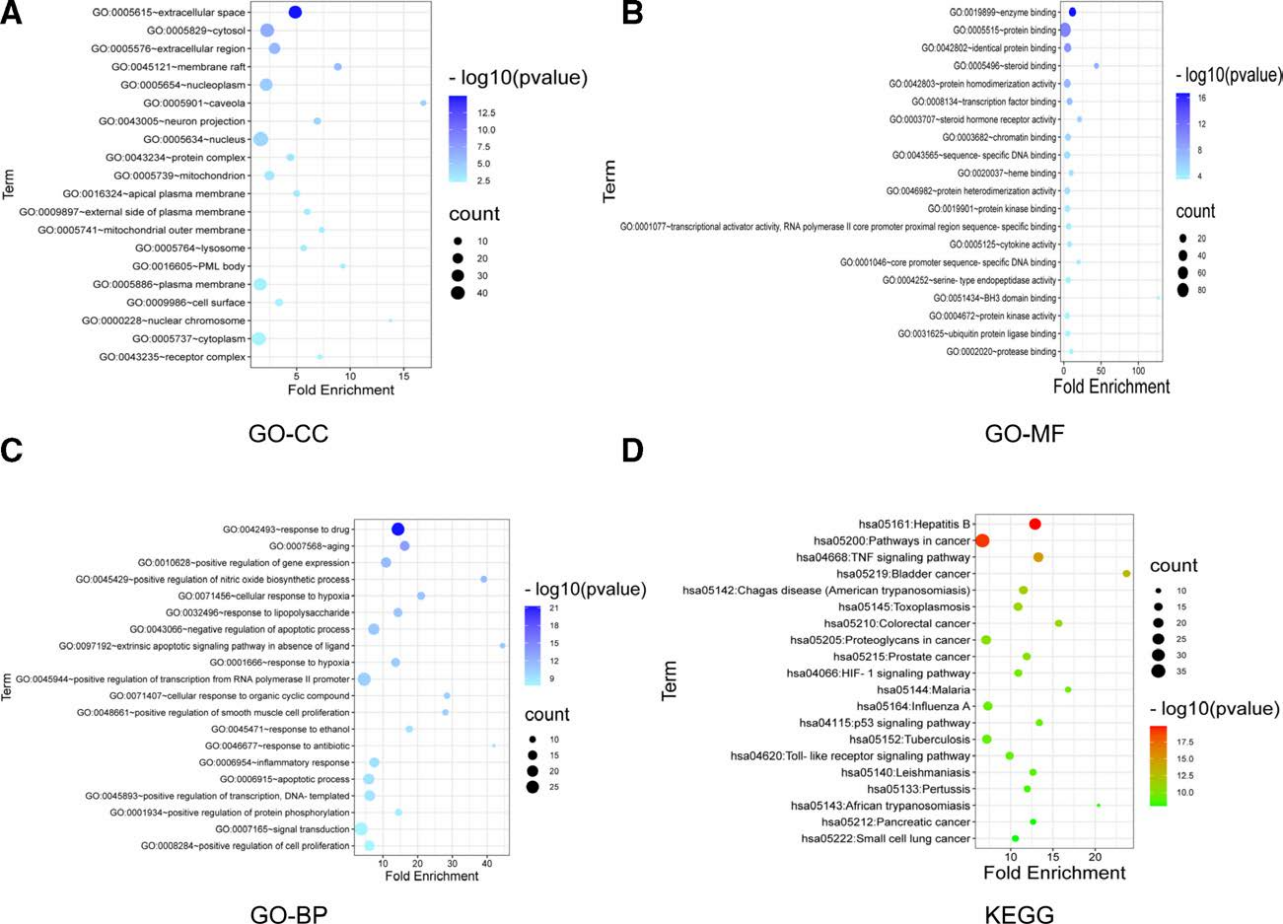
(A) Bubble diagram of GO-CC. (B) Bubble diagram of GO-MF. (C) Bubble diagram of GO-BP. (D) Bubble diagram of KEGG. BP = biological processes, CC = closeness centrality, GO = gene ontology, KEGG = Kyoto encyclopedia of genes and genomes.

The top 20 enrichment results of GO-CC were: extracellular space, cytosol, extracellular region, membrane raft, nucleoplasm, caveola, neuron projection, nucleus, protein complex, mitochondrion, apical plasma membrane, external side of plasma membrane, mitochondrial outer membrane, lysosome, PML body, plasma membrane, cell surface, nuclear chromosome, cytoplasm, receptor complex (Fig. 3A).

The top 20 enrichment results of GO-MF were: enzyme binding, protein binding, identical protein binding, steroid binding, protein homodimerization activity, transcription factor binding, steroid hormone receptor activity, chromatin binding, sequence-specific DNA binding, heme binding, protein heterodimerization activity, protein kinase binding, transcriptional activator activity, RNA polymerase II core promoter proximal region sequence-specific binding, cytokine activity, core promoter sequence-specific DNA binding, serine-type endopeptidase activity, BH3 domain binding, protein kinase activity, ubiquitin protein ligase binding, protease binding (Fig. 3B).

The top 20 enrichment results of GO-BP were: response to drug, aging, positive regulation of gene expression, positive regulation of nitric oxide biosynthetic process, cellular response to hypoxia, response to lipopolysaccharide, negative regulation of apoptotic process, extrinsic apoptotic signaling pathway in absence of ligand, response to hypoxia, positive regulation of transcription from RNA polymerase II promoter, cellular response to organic cyclic compound, positive regulation of smooth muscle cell proliferation, response to ethanol, response to antibiotic, inflammatory response, apoptotic process, positive regulation of transcription, DNA-templated, positive regulation of protein phosphorylation, signal transduction, positive regulation of cell proliferation (Fig. 3C).

**Figure 4.**
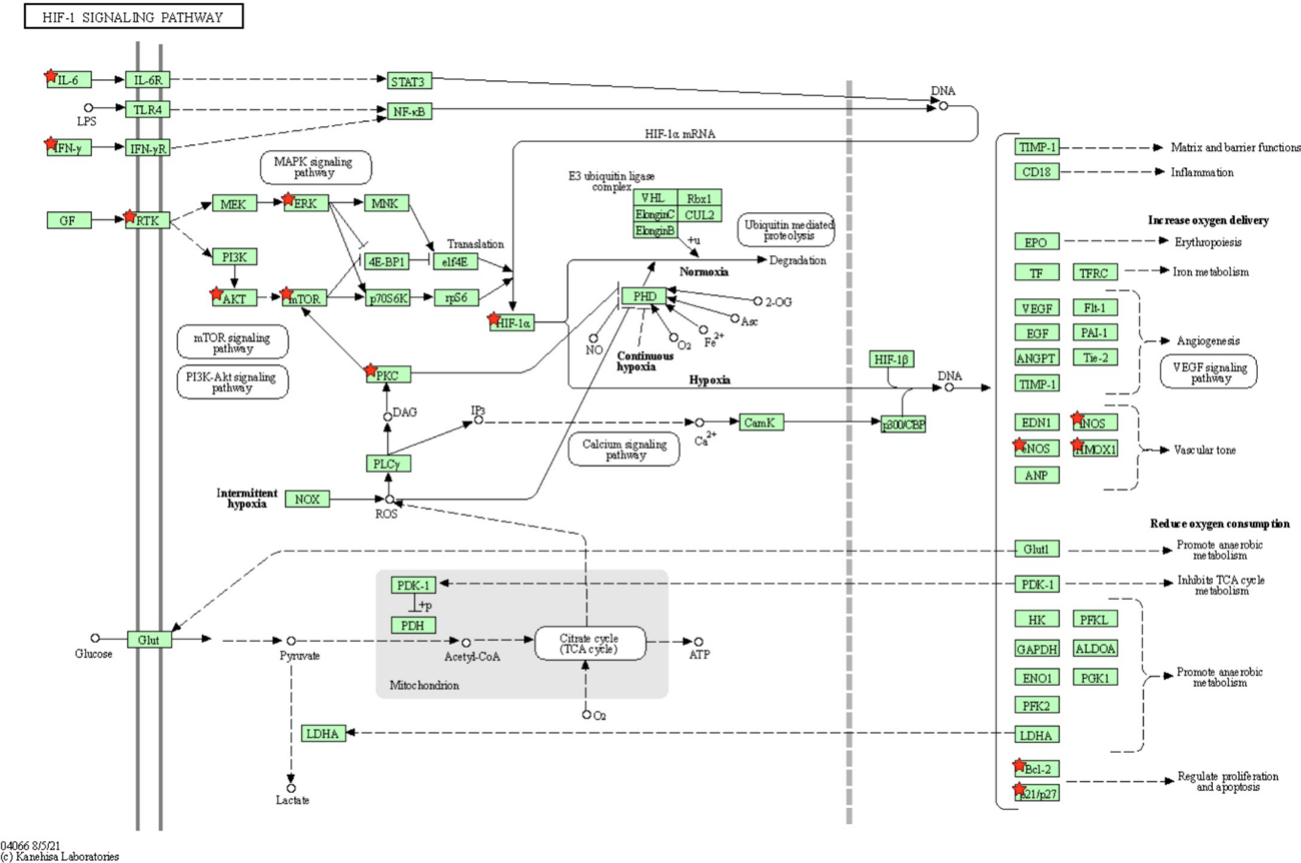
The HIF-1 signaling pathway (Note: Red pentagons are the interaction targets of Liuwei Dihuang Pill and tinnitus contained in the HIF-1 signaling pathway). HIF = hypoxia-inducible factor.

The top 20 KEGG enrichment results were Hepatitis B, Pathways in cancer, TNF signaling pathway, Bladder cancer, Chagas disease (American trypanosomiasis), Toxoplasmosis, Colorectal cancer, Proteoglycans in cancer, Prostate cancer, HIF-1 signaling pathway, Malaria, Influenza A, p53 signaling pathway, Tuberculosis, Toll-like receptor signaling pathway, Leishmaniasis, Pertussis, African trypanosomiasis, Pancreatic cancer, Small cell lung cancer (Fig. 3D). The HIF-1 signaling pathway is shown in the figure below (Fig. 4).

### 3.5. Construction of targets-pathways interaction network

**Figure 5.**
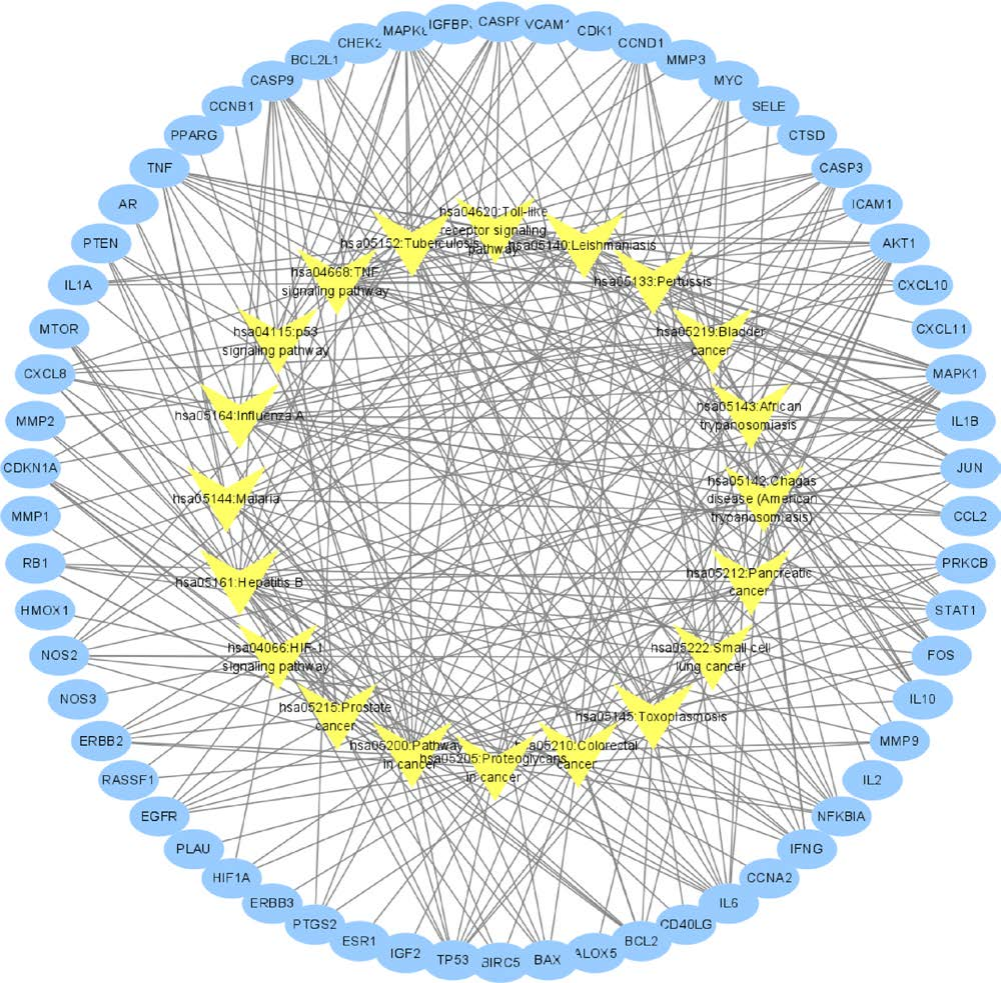
Targets-pathways interaction network (Note: Blue ovals represent the targets, yellow triangles represent the pathways).

The targets-pathways interaction network was constructed by Cytoscape software (Fig. 5).

### 3.6. Molecular docking results

**Figure 6.**
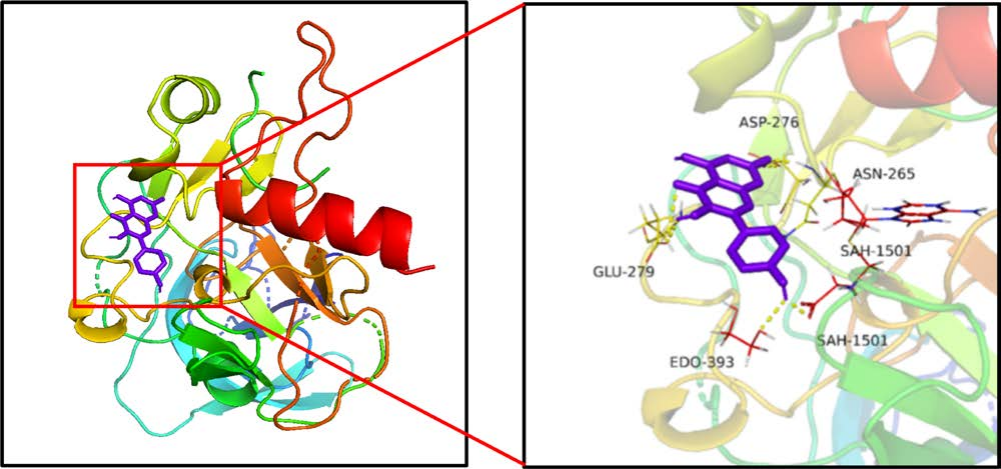
The docking mode of kaempferol and AKT1. AKT1 = serine/threonine kinase 1.

Molecular docking was carried out between the core components (quercetin, stigmasterol, kaempferol, β-sitosterol, and tetrahydroalstonine) and the core targets (TP53, JUN, MAPK1, AKT1 and TNF). The lower the binding free energy between the ligand and the receptor, the more stable the conformation is, which indicating the stronger the affinity of the 2.^[25]^ In general, when the binding free energy is less than 0, it indicates that the ligand and receptor can bind spontaneously.^[26]^ The binding energy results obtained by molecular docking in this study were shown (Table 2). The molecular docking results with the lowest binding free energy were visualized by PyMOL (Fig. 6).

**Table 2:** Molecular docking results.

Compounds	Target	PDB ID	Center(X, Y, Z)	Binding free energy(kcal/mol)
Quercetin	TP53	6WQX	28.998, −6.635, −14.746	−5.22
	JUN	3PTG	0.569, 0.171, 31.027	−3.56
	MAPK1	6G54	68.358, 15.167, 10.694	−4.72
	AKT1	3OS5	39.603, −18.581, −11.718	−4.17
	TNF	1FT4	19.583, −2.944, 24.173	−4.82
Stigmasterol	TP53	6WQX	28.998, −6.635, −14.746	−5.11
	JUN	3PTG	0.569, 0.171, 31.027	−4.59
	MAPK1	6G54	68.358, 15.167, 10.694	−4.26
	AKT1	3OS5	39.603, −18.581, −11.718	−6.04
	TNF	1FT4	19.583, −2.944, 24.173	−6.26
Kaempferol	TP53	6WQX	28.998, −6.635, −14.746	−4.68
	JUN	3PTG	0.569, 0.171, 31.027	−3.66
	MAPK1	6G54	68.358, 15.167, 10.694	−5.42
	AKT1	3OS5	39.603, −18.581, −16.173	−6.3
	TNF	1FT4	19.583, −2.944, 24.173	−4.64
β-sitosterol	TP53	6WQX	28.998, −6.635, −14.746	−4.18
	JUN	3PTG	0.569, 0.171, 31.027	−4.75
	MAPK1	6G54	68.358, 15.167, 10.694	−4.52
	AKT1	3OS5	39.603, −18.581, −11.718	−6.07
	TNF	1FT4	19.583, −2.944, 24.173	−6.01
Tetrahydroalstonine	TP53	6WQX	28.998, −6.635, −14.746	2.27
	JUN	3PTG	0.569, 0.171, 31.027	−4.29
	MAPK1	6G54	68.358, 15.167, 10.694	−3.23
	AKT1	3OS5	39.603, −18.581, −11.718	−6.01
	TNF	1FT4	19.583, −2.944, 24.173	−5.42

AKT1 = serine/threonine kinase 1, JUN = transcription factor AP-1, MAPK = mitogen-activated protein kinase, TNF = tumor necrosis factor, TP53 = tumor protein P53.

## 4. Discussion

### 4.1. Composition analysis of Liuwei Dihuang pill

In traditional Chinese medicine, there is a close relationship between the ear and the kidney, so nourishing the kidney and filling the essence is a common treatment for tinnitus, and Liuwei Dihuang pill can be selected by classics. Radix Rehmanniae Preparata has been used in traditional Oriental Medicine for the treatment of inner ear diseases, such as tinnitus and hearing loss. Fructus Corni also have been used for treatment of tinnitus. In the study of H. H. Yu, we investigated the protective effect of C. officinalis on hydrogen peroxide-induced cytotoxicity in auditory cells HEI-OC1.^[27]^ Y. Wang studied the prescription of Chinese medicine for tinnitus and found that Rhizoma Dioscoreae, Poria, Rhizoma Alismatis and Cortex Moutan are high-frequency medicines for tinnitus, especially suitable for tinnitus with deficiency of kidney essence.^[28]^

### 4.2. Analysis of the core components of Liuwei Dihuang pill in treating tinnitus

In this study, the core components of Liuwei Dihuang Pill in the treatment of tinnitus were preliminarily screened by the methods of traditional Chinese medicine network pharmacology and molecular docking, which were quercetin, stigmasterol, kaempferol, β-sitosterol, tetrahydroalstonine and so on. Quercetin is a flavonoids compound with antioxidant, anti-inflammatory, antitussive, antiasthmatic, and antiallergic effects. Hearing loss usually gives rise to subjective tinnitus, a phantom ringing sensation that occurs in the absence of external sounds, resulting from a compensatory increase in gain or neural amplification in the central auditory system to compensate for the loss of sensory input from the cochlea. The inner ear is highly susceptible to ischemia and oxidative damage, Current therapeutic strategies have proven that the goal of the traditional oriental medicine principle of increase humoral principles is a relevant approach for reducing the development of hearing loss by improving microcirculation in the blood-labyrinth barrier and increasing cochlear blood flow. The potential benefits of traditional oriental medicine drugs extend to a multi-target approach to different auditory structures of the inner ear that are associated with increased cochlear blood flow, antioxidant, anti-inflammatory, anti-apoptotic and neuroprotective activities. Studies have shown that about 25 herbs and 40 active compounds in traditional oriental medicine are used in the treatment of hearing loss and tinnitus, which include quercetin. Both in vitro and in vivo experiments have shown beneficial effects on acquired sensorineural hearing loss caused by external noise, aging, ototoxic drugs, or diabetes.^[29]^ Y. Hirose et al experimentally investigated whether quercetin could prevent hair cell loss in the zebrafish lateral line and guinea pig cochlea, which found that quercetin reduced antioxidant activity and protected the cochlea from noise exposure in rodent.^[30]^ T. Yang found that quercetin could significantly prevent gentamicin-induced hair cell damage by reducing ROS and NO formation.^[31]^ Stigmasterol has anti-inflammatory, anti-oxidant, anti-cancer, and cholesterol-lowering effects,^[32]^ and can be extracted from bell grass and other active ingredients to treat deafness and tinnitus.^[33]^ J. Li screened active ingredients for the prevention and treatment of deafness based on cell and zebrafish injury models, and found that stigmasterol had a preventive effect on cisplatin-induced zebrafish hair cell damage.^[34]^ Kaempferol has antioxidant and antiapoptotic properties, protecting cells from cisplatin-induced apoptosis in a dose-dependent manner in HEI-OC1 cells.^[35]^ β-Sitosterol has antioxidant, cholesterol-lowering, anti-inflammatory, immunomodulatory, and anti-tumor effects.^[36]^ R. Paniagua-Pérez et al found a mean inhibition of inflammatory inhibition of 75% by β-sitosterol in the mouse ear edema test.^[37]^

### 4.3. Analysis of core targets of Liuwei Dihuang pill in the treatment of tinnitus

In this study, the core targets TP53, JUN, MAPK1, AKT1 and TNF were obtained according to the PPI network. Hearing loss and tinnitus induced by aspirin and sodium salicylate are considered to be completely reversible problems that disappear within a few days after discontinuation use, and the 3 genes with the greatest increase in apoptotic gene expression all belong to the TNF family or its corresponding receptor genes, the tumor suppressor gene P53, was also significantly up-regulated (2.00-fold).^[38]^ J. Zhai explored the effects of serum inflammatory factors and neurotrophic factors of the cochlear tissue of presbycusis in rats and found that presbycusis is closely related to TNF-α and other serum inflammatory factors in cochlear tissue, and its abnormal expression in the cochlea of aged rats is of great significance for the diagnosis, treatment and prognosis of presbycusis.^[39]^ Studies have shown that tumor necrosis factor alpha (TNF-α) expression is elevated by loud noise exposure in the auditory periphery and cochlear nucleus and is associated with tinnitus, and pharmacological blockade of TNF-α expression prevents noise-induced neuroinflammation and tinnitus. Conversely, infusion of TNF-α into primary auditory cortex resulted in behavioral signs of tinnitus in wild-type and TNF-α knockout mice with normal hearing.^[40]^

### 4.4. Analysis of signaling pathway of Liuwei Dihuang pill in the treatment of tinnitus

The HIF-1 signaling pathway, Influenza A, p53 signaling pathway, Toll-like receptor signaling pathway, Hepatitis B, TNF signaling pathway, Toxoplasmosis, etc. The results obtained by enrichment analysis are all closely related to tinnitus, mainly related to anti-inflammatory, improving the microcirculation of the blood labyrinth barrier, increasing cochlear blood flow, reducing hair cell damage, and so on. Mechanisms associated with the development of tinnitus and acquired hearing impairment include hair cell loss, signal transduction disturbances in the region of the outer and inner hair cells and the spiral ganglion, impaired cochlear blood flow, mechanical impairment, and hypoxia and ischemia.^[41]^ Hypoxia-inducible factor (HIF) is a key mediator of adaptation to hypoxia, enhancing transcription of various target genes, reducing oxygen-dependent pathways by regulating metabolism.^[42]^ Transcription factor HIF-1 (hypoxia-inducible factor-1) regulates the expression of genes involved in glucose supply, growth, metabolism, redox reactions and blood supply. Hypoxia and ischemia play an important role in the pathogenesis of tinnitus and hearing loss.^[43]^ B. Mazurek investigated the possible cellular and molecular biological causes of peripheral hearing loss and tinnitus, discussed the role of hypoxia and ischemia in the cochlea and in the etiology of the neurosensory types of tinnitus, and found that HIF-1 may act as a key transcription factor play important roles in cellular adaptation to hypoxia. TNF plays a regulatory role in reducing, maintaining and improving inflammatory responses, and Riva C et al also explored auditory function in cd/1 mice at 4, 12, and 24 weeks of age and correlated it with the presence of oxidative damage in the cochlea and the regulation of cochlear HIF-1 responsive target genes, associated with multiple inflammatory pathways such as TNF-α, or cell death with p53 protein, Bax protein and surviving factors with insulin-like growth factor-1.^[44]^ Furthermore, hypoxia modulates molecular processes in both acute and chronic tinnitus. While under hypoxic/ischemic conditions, HIF-1 induces changes in gene expression that may contribute to the remodeling of specific structures within the cochlea.^[45]^ p53 is a key component of signaling networks that protect cells from various stresses. Studies have shown that the Mdm2/p53 interaction has an important role in ensuring the survival of proliferative progenitor cells and differentiated cells of the auditory organ.^[46]^ H. Xiong show that activation of miR-34a/SIRT1/p53 signaling contributes to cochlear hair cell apoptosis, and has implications for age-related hearing loss.^[47]^ Changes in the upregulated modulator of apoptosis p53 directly or within the apoptosis-related cascade may play an important role during development, especially in the outer ear, where TP53 mRNA expression was significantly downregulated in patients with ear atresia compared to controls.^[48]^ Tlr4 regulates multiple aspects of the immune response in the cochlea and contributes to cochlear pathogenesis after acoustic damage.^[49]^ C. Yang showed that higher expression of the TLR2 gene was found in patients with profound hearing loss compared to those with milder hearing loss (*P* < .05).^[50]^ G. T. Van Well also found that the TLR system appears to play an important role in the immune response to the BM and subsequent neuronal damage and cochlear inflammation.^[51]^ In addition, the foreign literature reports that the hepatitis B vaccine and Toxoplasma gondii can cause acute tinnitus， sudden deafness, and other diseases.^[52,53]^ Epidemiological studies have found many common respiratory diseases such as influenza A in the middle ear effusion of children with acute otitis media.^[54]^

## 5. Conclusions

Based on network pharmacological analysis, this study demonstrated that Liuwei Dihuang Pill treated tinnitus through multi-compounds, multi-targets, and multi-pathways, and preliminarily clarified the related potential mechanism of Liuwei Dihuang Pill in the treatment of tinnitus. Through KEGG pathway enrichment analysis, it was found that Liuwei Dihuang Pill played an important role in the treatment of tinnitus, including HIF-1 signaling pathway, Influenza A, P53 signaling pathway, and Toll-like receptor signaling pathway, which might play a role in anti-inflammatory, improving microcirculation in the blood-labyrinth barrier, increasing cochlear blood flow, and preventing hair cell damage. Although subsequent validations are needed to determine the exact mechanism of Liuwei Dihuang Pill, our present study provide promising directions for future research.

## Acknowledgments

The authors would like to thank Jiangxi Hospital of Integrated Traditional Chinese and Western Medicine, Affiliated Hospital of Jiangxi University of Traditional Chinese Medicine, Jiangxi University of Traditional Chinese Medicine, and Pinghu Hospital of Traditional Chinese Medicine. This paper is supported by State Administration of Traditional Chinese Medicine (Grant number: 201507006. Project name: Special Scientific Research project of TCM Industry of State Administration of Traditional Chinese Medicine), and Jiangxi Provincial Health and Family Planning Commission (Grant number:2018A375. Project name: Special Scientific Research project of TCM Industry of State Administration of Traditional Chinese Medicine). The authors are grateful to Mr. Yulong Ji for his help with the preparation of this paper. We would like to thank the editors and reviewers for their helpful remarks that improved this paper.

## Author contributions

**Conceptualization:** Zhongbiao Wu, Zhongyan Zhu.

**Data curation:** Zhongbiao Wu, Zhongyan Zhu, Xinmei Huang.

**Formal analysis:** Zhongbiao Wu, Zhongyan Zhu, Weikun Wu.

**Funding acquisition:** Zhongbiao Wu, Chengcheng Deng, Qiang Xie.

**Investigation:** Jian Cao, Chengkun You.

**Methodology:** Jian Cao, Weikun Wu, Shiping Hu, Chengkun You.

**Project administration:** Zhongbiao Wu, Zhongyan Zhu.

**Resources:** Jian Cao, Weikun Wu, Xinmei Huang.

**Software:** Zhongyan Zhu, Shiping Hu.

**Supervision:** Zhongbiao Wu, Zhongyan Zhu.

**Validation:** Shiping Hu, Chengcheng Deng, Qiang Xie.

**Visualization:** Chengcheng Deng, Qiang Xie.

**Writing – original draft:** Chengcheng Deng, Qiang Xie, Xinmei Huang, Chengkun You.

**Writing – review & editing:** Xinmei Huang, Chengkun You.
